# Emerging mechanisms and functions of inflammasome complexes in teleost fish

**DOI:** 10.3389/fimmu.2023.1065181

**Published:** 2023-02-16

**Authors:** Ming Xian Chang

**Affiliations:** ^1^ State Key Laboratory of Freshwater Ecology and Biotechnology, Institute of Hydrobiology, Chinese Academy of InSciences, Wuhan, China; ^2^ College of Advanced Agricultural Sciences, University of Chinese Academy of Sciences, Beijing, China; ^3^ Innovation Academy for Seed Design, Chinese Academy of Sciences, Wuhan, China

**Keywords:** canonical inflammasomes, noncanonical inflammasomes, sensors, ASC, inflammasome-associated effectors, bacterial infection

## Abstract

Inflammasomes are multiprotein complexes, which are assembled in response to a diverse range of exogenous pathogens and endogenous danger signals, leading to produce pro-inflammatory cytokines and induce pyroptotic cell death. Inflammasome components have been identified in teleost fish. Previous reviews have highlighted the conservation of inflammasome components in evolution, inflammasome function in zebrafish infectious and non-infectious models, and the mechanism that induce pyroptosis in fish. The activation of inflammasome involves the canonical and noncanonical pathways, which can play critical roles in the control of various inflammatory and metabolic diseases. The canonical inflammasomes activate caspase-1, and their signaling is initiated by cytosolic pattern recognition receptors. However the noncanonical inflammasomes activate inflammatory caspase upon sensing of cytosolic lipopolysaccharide from Gram-negative bacteria. In this review, we summarize the mechanisms of activation of canonical and noncanonical inflammasomes in teleost fish, with a particular focus on inflammasome complexes in response to bacterial infection. Furthermore, the functions of inflammasome-associated effectors, specific regulatory mechanisms of teleost inflammasomes and functional roles of inflammasomes in innate immune responses are also reviewed. The knowledge of inflammasome activation and pathogen clearance in teleost fish will shed new light on new molecular targets for treatment of inflammatory and infectious diseases.

## Highlights

1. NLRP1 and NLRP3 inflammasomes exist in teleost fish.
2. Inflammasome components or inflammasome-associated effectors may vary on the fish species selected.
3. The specific regulatory mechanisms of inflammasome activation exist in teleost fish.
4. The activation mechanisms of inflammasome-associated effectors may vary on different pathogens.

## Introduction

Inflammasomes are cytosolic protein complexes which can recognize various exogenous pathogens and endogenous danger signals. Formation of a functional inflammasome is initiated by germline-encoded pattern recognition receptors (PRRs). In response to pathogen-associated molecular patterns (PAMPs), danger-associated molecular patterns (DAMPs) or homeostasis-altering molecular processes (HAMPs), certain PRRs recruit the adaptor protein called apoptosis-associated speck-like protein (also known as ASC or PYCARD) and an effector pro-caspase-1 to form the functional canonical inflammasome (1). Inflammasome complexes are assembled *via* homotypic CARD (caspase activation and recruitment domain)–CARD or PYD (pyrin domain)–PYD interactions. Among several families of PRRs, the nucleotide-binding domain and leucine-rich repeat receptors (NLRs), the absent in melanoma 2-like receptors (ALRs) and pyrin have been described to form the expanding inflammasome family, which includes the NLR family pyrin domain containing 1 (NLRP1), 3 (NLRP3), 6 (NLRP6), 7 (NLRP7), 9b (NLRP9b), NLR-family CARD-containing protein 4 (NLRC4) and 5 (NLRC5), AIM2, IFI16 and pyrin canonical inflammasomes (1–3). Different from these canonical inflammasomes mentioned above, which convert pro-caspase-1 into the catalytically active caspase-1, the non-canonical inflammasome promotes caspase-11 activation in mice or caspase-4 and caspase-5 activation in human (2, 4, 5).

Caspase-1 activation leads to cleave the proinflammatory cytokines pro-IL-1β and pro-IL-18 into their mature forms IL-1β and IL-18, and also activates the proteolytic cleavage of the gasdermin D (GSDMD) to induce pyroptosis, a form of cell death (6, 7). Activation of caspases 4, 5 and 11 also leads to pyroptosis (6). Generally, the secretion of IL-1β and IL-18 and the activation of inflammatory cell death contribute to antimicrobial defense, and inflammasome activation plays beneficial roles for the host against pathogen infection. Whereas aberrant activation and regulation of inflammasome triggered by PAMPs or DAMPs may lead to the pathogenesis of autoimmune, metabolic or neurodegenerative diseases (8–11). Therefore, inflammasomes act as a double-edged sword with both protective and detrimental potential for host health.

Zebrafish have become a research model to study human diseases due to a wide range of advantages including: (i) high fecundity and rapid embryonic development; (ii) transparent embryos and the ability easy for gene knockdown or overexpression; (iii) high quality of genome assembly and up to 70% of human genes that have at least one obvious zebrafish orthologue (12, 13). Previous reviews have highlighted inflammasome components in zebrafish, including inflammasome sensors (such as NLRP6, GBP1, GBP3, GBP4, NLR-B30.2 genes), inflammasome adaptors (ASC and Caiap), proinflammatory caspases (caspa, caspb, caspbl and caspc) and inflammasome effector GSDMEb, and discussed the activation of inflammasomes during pathogenic infection and the induction of pyroptosis in teleost fish (14, 15). Different from previous reviews, the current review will focus on the recent research advances made in terms of NLRP1 inflammasome, NLRP3 inflammasome, NLRC4 inflammasome and non-canonical inflammasome (**Table 1**), with a particular emphasis on the mechanisms that regulate inflammasome signaling as well as functional roles of piscine inflammasome-associated effectors and inflammasomes in innate immune responses.

**Table 1:** Summary of inflammasomes in teleost fish.

Inflammasome	Research model	Activator of inflammasome	Mechanism	Function	Ref.
NLRP1	common carp	MDP	/	/	(16)
	zebrafish	bacterial LPS, MDP and DNA	ASC-dependent way; sequential activation of caspy and caspy2	Antibacterial innate immunity against *E. tarda* infection	(17)
NLRP3	zebrafish	LPS and cellular oxidation	ASC-dependent way; sequential activation of caspy/caspy2	ASC-dependent IL-1β maturation; GSDME–mediated pyroptosis	(18)
	carp	Cd	/	Inducing lymphocytes pyroptosis	(19)
	Japanese flounder	nigericin, ATP or MSU	ASC-dependent way; activation of JfCaspase-1	Controlling IL-1β production; defensing against *E. piscicida* invasion	(20)
	turbot	nigericin, ATP or MSU	ASC-dependent way; activation of SmCaspase	Bacterial clearance of *E. piscicida*; epithelial desquamation in gill filaments	(21)
NLRC4	zebrafish	Lm-pyro *L. monocytogenes*, a strain engineered to activate the inflammasome via ectopic expression of flagellin	/	Inducing macrophage recruitment to infection sites; controlling *L. monocytogenes* infection	(22)
Non-canonical inflammasome	zebrafish	LPS	caspy2 binds directly to LPS, resulting in caspy2 oligomerization	Restricting bacterial invasion of *E. piscicida*; NETosis	(23, 24)
	turbot	LPS	SmCaspase directly binds with LPS	Bacterial clearance of *E. piscicida*	(21)

## NLRP1 inflammasome

NLRP1, which is also known as NACHT-leucine-rich-repeat protein-1 (NALP1), belongs to the superfamily of NLRs. The human NLRP1 is structurally characterized by the presence of an N-terminal PYD, a nucleotide-binding domain (NBD or NACHT), a leucine-rich repeat (LRR) domain, a ‘function to find’ (FIIND) domain, and a C-terminal CARD (25). NLRP1-encoding genes are found in most mammalian species, and undergo extensive diversification among different species (26). Humans and most primates encode only a single NLRP1 paralog, however there are 3 paralogs of NLRP1 in mice, namely NLRP1a, NLRP1b and NLRP1c. Different from human NLRP1, mice NLRP1 paralogs contain NR100, NACHT, LRR, FIIND and CARD domains (27). The mammalian NLRP1 auto-cleaves for generating an N-terminal and a C-terminal fragment (28). Murine NLRP1 inflammasome is assembled by recruitment of caspase-1 independently of ASC (29). However for human NLRP1 inflammasome, the C-terminal fragment of NLRP1 containing the partial FIIND and the entire CARD recruits ASC to activate caspase-1 through CARD–CARD instead of PYD–PYD interactions (28).

Mammalian NLRP1 inflammasome can be activated by muramyl dipeptide (MDP), reduction of cytosolic ATP or pathogens such as *Shigella flexneri*, *Toxoplasma gondii* and *Listeria monocytogenes* (25, 30, 31). The activation of NLRP1 inflammasome is also regulated by diverse pathogen-encoded enzymatic activities. The IpaH7.8 ubiquitin ligase secreted by the type III secretion system (T3SS) from *S. flexneri* and the lethal factor (LF) protease secreted by *Bacillus anthracis* activate NLRP1b inflammasome *via* “functional degradation” (32, 33). The protease from tobacco etch virus and the virally encoded 3C protease termed 3C^pro^ from diverse picornaviruses can specifically cleave human NLRP1, leading to activation of the NLRP1 inflammasome (28, 34). Furthermore, the inhibition of the cytosolic dipeptidyl peptidases DPP8 and DPP9 by Val-boroPro inhibitor activates the murine NLRP1b inflammasome (35). Human DPP9 has been confirmed to be a specific NLRP1-interacting partner and function as an endogenous inhibitor of NLRP1 inflammasome (36).

**Figure 1:**
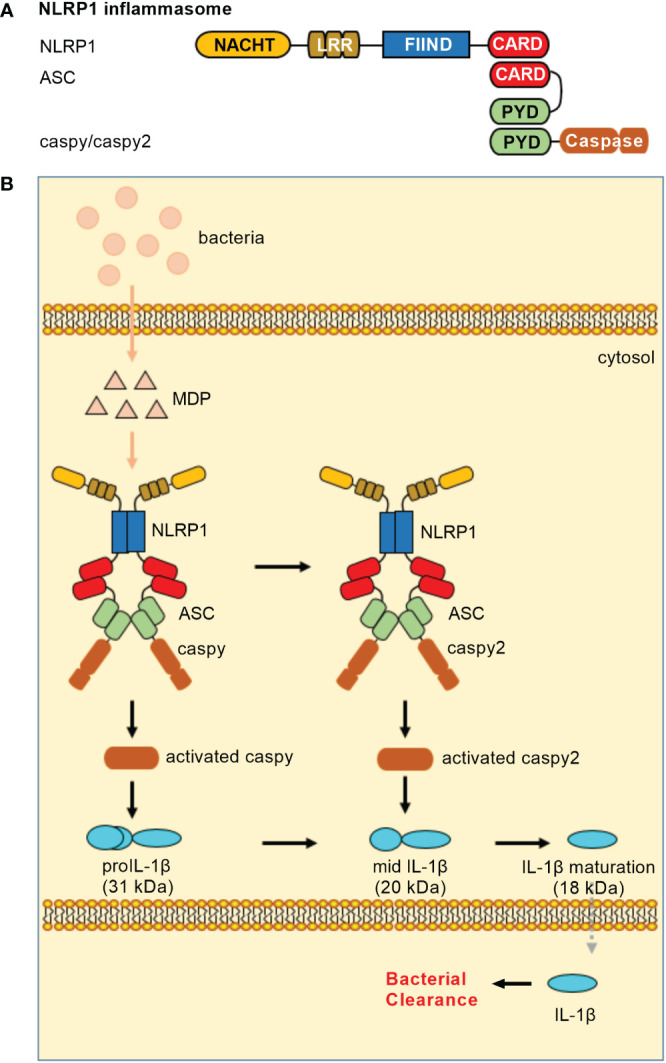
NLRP1 inflammasomes in teleost fish. **(A)** Minimal NLRP1 inflammasomes. Domains: NACHT, nucleotide-binding and oligomerization domain; LRR, leucine-rich repeat; FIIND, domain with function to find; CARD, caspase recruitment domain; PYD, pyrin domain. **(B)** Activation of the NLRP1 inflammasome. Teleost NLRP1 inflammasome complex formation was triggered by MDP but not by LPS.

In teleost, NLRP1 inflammasome is only reported in common carp (*Cyprinus carpio*) and zebrafish. The piscine NLRP1 possesses NACHT, LRR, FIIND and CARD domains (16, 17), however lacks PYD in the N-terminal. In zebrafish, caspase-a (caspy) and caspase-b (caspy2), whose N-terminal regions share higher sequence similarity with the PYD than the CARD of mammalian caspases, are analogues of mammalian caspase-1 and caspase-4/5, respectively (37). Zebrafish NLRP1 containing a CARD cannot directly bind with caspy and caspy2 without a CARD. The recruitment of caspy and caspy2 into zebrafish NLRP1 inflammasome needs the help of ASC. Furthermore, sequential activation of caspy and caspy2 in zebrafish NLRP1 inflammasome for the maturation of IL-1β is dependent on the caspy– and caspy2–directed cleavage order, with a preference for caspy and a subsequent choice for caspy2. In short, the domain organization of zebrafish NLRP1 is the same as mice NLRP1. However, the mechanism of zebrafish NLRP1 in triggering the activation of inflammatory caspase and maturation of IL-1β is in an ASC-dependent way (**Figure 1**), which is different from murine NLRP1 inflammasome that activates caspase-1 in an ASC-independent way (38).

Similar to mammalian NLRP1 inflammasome, MDP has been confirmed to activate piscine NLRP1 inflammasome (16, 17). In zebrafish, it is also suggested that the activation of NLRP1 inflammasome may be promoted by the alteration of redox state during bacterial infection. In response to *Edwardsiella tarda* infection, zebrafish NLRP1 inflammasome played important roles in antibacterial immune response, with the high mortality observed for zebrafish NLRP1 morphants (17). However, whether pathogen-encoded effectors activate piscine NLRP1 inflammasome *via* ‘functional degradation’ is unclear. Future research is needed to clarify regulatory mechanisms for NLRP1 inflammasome in teleost.

## NLRP3 inflammasome

Among numerous inflammasomes identified in mammals, NLRP3 inflammasome consisting of NLRP3, ASC and caspase-1 is the most extensively studied inflammasome complex. The mammalian NLRP3 is structurally characterized by the presence of the amino-terminal PYD for homotypic interactions with the ASC, the central NACHT domain for facilitating self-oligomerization, and the C-terminal LRRs domain for sensing endogenous alarmins and microbial ligands (39). Upon stimulation, NLRP3 firstly self-oligomerizes through its NACHT domain, and then recruits ASC through its PYD for nucleating helical ASC clusters. Multiple ASC filaments coalesce into an ASC speck, and the assembled ASC nucleates caspase-1 filaments through CARD-CARD interactions (40, 41).

Several cellular signaling events for activating the NLRP3 inflammasome, which include ion flux, mitochondrial damage and dysfunction, and lysosomal disruption, have been observed (42, 43). To date, studies show that various pathogens, PAMPs, DAMPs or environmental irritants, such as *Candida albicans*, influenza virus, LPS, lipooligosaccharide, MDP, nucleic acids, pore-forming toxins, asbestos, silica, nanoparticles, aluminum hydroxide, cholesterol crystals, ATP, monosodium urate (MSU), hyaluronan, and heparan sulfate so on, activate the NLRP3 inflammasome by inducing canonical, noncanonical or alternative NLRP3 inflammasome (44, 45). Canonical NLRP3 inflammasome activation requires the priming and activation two steps. The ligands of toll-like receptors (TLRs), NLRs and cytokine receptors induce the expression of pro-IL-1β and NLRP3 *via* the myd88-NF-κB pathway during the priming step, whereas PAMPs or DAMPs promote NLRP3 inflammasome assembly during the activation step (46). Different from canonical NLRP3 inflammasome activation, noncanonical NLRP3 inflammasome activation occurs downstream of caspase-11, which is initiated by cytosolic LPS (47). For alternative NLRP3 inflammasome activation, TLR ligands alone are sufficient to activate the NLRP3 inflammasome *via* the TLR4–TRIF–RIPK1–FADD–CASP8 signaling axis, which is absence of classical inflammasome characteristics including inducing K+ efflux, pyroptosome formation or pyroptosis (48).

**Figure 2:**
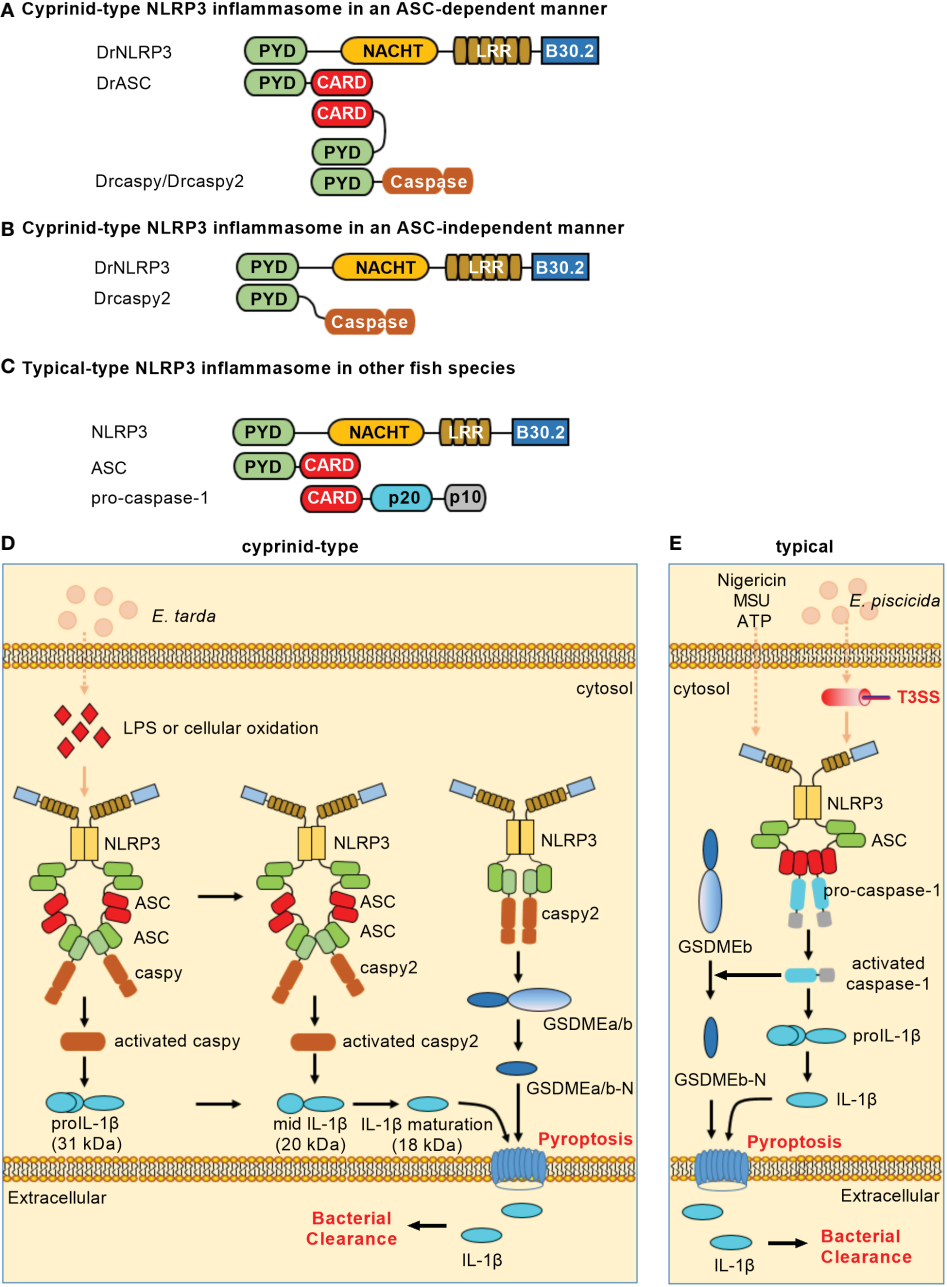
NLRP3 inflammasomes in teleost fish. **(A)** Minimal NLRP3 inflammasomes in an ASC-dependent manner in cyprinids. **(B)** Minimal NLRP3 inflammasomes in an ASC-independent manner in cyprinids. **(C)** Minimal NLRP3 inflammasomes in other fish species. For **(A–C)**, domains are shown as follows: PYD, pyrin domain; NACHT, nucleotide-binding and oligomerization domain; LRR, leucine-rich repeat; CARD, caspase recruitment domain. **(D)** Activation of the NLRP3 inflammasome in cyprinids. The ASC-dependent NLRP3 inflammasome contributes to caspy/caspy2 activation, which leads to proIL-1β maturation. Meanwhile, NLRP3 possesses the ability to directly recruit and activate caspy2, which is sufficient for cleaving GSDMEa/b to elicit pyroptosis. **(E)** Activation of the NLRP3 inflammasome in other fish species. In the most fish such as Japanese flounder and turbot, NLRP3, ASC and pro-caspase-1 form the canonical NLRP3 inflammasome. The activation of caspase-1 can recognize and cleave the GSDMEb to release its N-terminal domain, mediating pyroptosis and restricting bacterial infection against *Edwardsiella piscicida*.

In teleost fish, NLRP3 inflammasomes have been identified in zebrafish (18), carp (19), Japanese flounder (*Paralichthys olivaceus*) (20) and turbot (*Scophthalmus maximus*) (21). Similar to the domain architecture of mammalian NLRP3, piscine NLRP3 contains an N-terminal PYD, a central NACHT and a series of LRRs. Besides the three domains, piscine NLRP3 still contains a C-terminal B30.2 (PRY/SPRY) domain that is unique in fish (18, 20). However, the B30.2 domain is not functional in the assembly of NLRP3 inflammasome and the activation of caspy/caspy2. Cyprinid-type NLRP3 inflammasome consists of NLRP3, ASC and caspy/caspy2 (**Figure 2A**) or of NLRP3 and caspy2 (**Figure 2B**). In other fish species except Cyprinidae, NLRP3 inflammasome consists of NLRP3, ASC and pro-caspase-1 (**Figure 2C**). Upon ligand recognition, the PYD of NLRP3 interacts with the PYD of ASC. Then, the recruitment and activation of caspase-1 lead to the ASC-dependent IL-1β maturation and GSDME-mediated pyroptosis. Cyprinid-type NLRP3 also directly activates caspy2 and elicits cell pyroptosis in a GSDME-dependent but ASC-independent manner (18).

LPS and cellular oxidation were found to contribute to the activation of zebrafish NLRP3 inflammasome. The knockdown of zebrafish NLRP3 increased the mortality of embryos with *E. tarda* infection (18). In the HKM cells of Japanese flounder, ATP, nigericin or MSU could trigger the NLRP3 inflammasome. In response to *E. piscicida* infection, knockdown of either NLRP3 or caspase-1 promoted the bacterial colonization, while overexpression of NLRP3 or caspase-1 hampered the bacterial colonization in Japanese flounder (20). These studies reveal the importance of teleost NLRP3 inflammasome in restricting bacterial infection *in vivo*. Besides PAMPs and DAMPs, the piscine NLRP3 inflammasome can also activated by toxic metals such as Cadmium (Cd). The exposure of Cd can induce pyroptosis of lymphocytes by activating NLRP3 in carp pronephros and spleens, and inhibition of NLRP3 activity can slow down the degree of lymphocytes pyroptosis (19). However in sharp contrast, the activators of mammalian NLRP3 inflammasomes, including ATP, nigericin, cell swelling, MSU and aluminum fail to activate caspase-1 and IL-1β processing in seabream macrophages, although stimulation of macrophages with PAMPs or DAMPs promoted the processing of proIL-1β and the release of IL-1β (49). These data suggest that although the mechanisms involved in IL-1β secretion are conserved throughout evolution, the activators of NLRP3 inflammasome or inflammasomes selected used for the secretion of IL-1β may varied on different fish species (**Table 1**).

Zebrafish NLRP3 recruits ASC and caspy/caspy2 to mediate IL-1 maturation for triggering canonical inflammasome activation. Similar to the NLRP1 inflammasome, the sequential activation of caspy/caspy2 is also observed in an NLRP3 inflammasome. Meanwhile, zebrafish NLRP3 directly recruits and activates caspy2 to induce IL-1β secretion and cell pyroptosis in a GSDME-dependent manner for triggering noncanonical NLRP3 inflammasome activation (18). Moreover, the NLRP3 from Japanese flounder can assemble ASC through PYD-PYD interaction and trigger caspase-1 activation and IL-1β maturation (20). In the turbot, the inflammatory caspase (SmCaspase) can directly recognize LPS through its N-terminal CARD and possess a caspase-5-like substrate specificity. However different from its mammalian or zebrafish counterparts, SmCaspase can associate with NLRP3-ASC complex to mediate canonical NLRP3 inflammasome activation (21). The mechanisms of NLRP3 inflammasome activation in teleost fish are depicted in **Figures 2D, E**. Whether alternative NLRP3 inflammasome activation exists in teleost fish remains unknown.

## NLRC4 inflammasome

NLRC4 was initially described as a pro-apoptotic protein, which could specifically activate caspase-1 *via* CARD-CARD interaction and induce apoptosis in human cells in a caspase-1-dependent manner (50). NLRC4, which contains an N-terminal CARD, a central NACHT domain and C-terminal LRRs, is a critical component of defense against enteric or systemic pathogens (51). Several pathogens such as *Salmonella Typhimurium*, *Burkholderia pseudomallei*, *Escherichia coli*, *S. flexneri* and *Pseudomonas aeruginosa* possess flagellin-like virulence factors and can activate the NLRC4 inflammasome (52). Besides bacterial flagellin, NLRC4 inflammasome is also activated by the type III and type IV secretion systems of bacteria (53, 54).

NAIPs, which contain an N-terminal baculovirus IAP-repeat (BIR) domain, a central NACHT, and a carboxy-terminal LRR, act as upstream sensors for NLRC4 inflammasome assembly (55). Upon interaction of inactive NAIP proteins with ligands and ligand activation of NAIP proteins, NAIP changes its conformation and associates with NLRC4 to induce activation of the NAIP/NLRC4 inflammasome (56). The CARD of NLRC4 allows it to directly bind to the CARD of caspase-1, which is different from other inflammasome proteins that need the adaptor protein ASC. However NLRC4 can also bind ASC, leading to more efficient activation of caspase-1 and cytokine secretion (57). Furthermore, a study showed that WD repeat containing protein 90 (WDR90) is a new component of the NLRC4 inflammasome in human. WDR90 could interact with NLRC4, and specifically mediate the cellular redistribution of NLRC4, but not for NLRP3 and AIM2 (58).

Compared to the mammalian NAIP/NLRC4 inflammasome, little is known about this inflammasome in other species. The murine genome encodes seven NAIP proteins. Among them, NAIP1 and NAIP2 sense needle and inner rod protein of the Type 3 secretion system respectively, and NAIP5/NAIP6 for flagellin (59, 60). Although humans only encode one single NAIP protein (hNAIP), the hNAIP could sense needle, inner rod protein and flagellin (61, 62). Interestingly, pigs have a single locus encoding NLRC4 and NAIP, but neither the NLRC4 nor the NAIP gene was expressed in pigs, which suggest that pigs lack the NLRC4 inflammasome (63). In zebrafish larvae, WDR90 is involved in caspy activation, and acts upstream of ASC and caspy to promote *S. Typhimurium* resistance (58). The virulence effector trxlp of *E. piscicida*, which is an important pathogenic bacterium that causes hemorrhagic septicemia in fish, mainly promotes the NLRC4 but not NLRP3 inflammasome activation during *E. piscicida* infection in murine macrophages (64). In response to the infection of Lm-pyro *L. monocytogenes*, a strain engineered to activate the inflammasome *via* ectopic expression of flagellin, zebrafish inflammasome is activated, which leads to the recruitment of macrophages to infection sites and confers host protection to bacterial infection. These data suggest that this signaling axis similar to mammalian NLRC4 inflammasome sensing flagellin is present in zebrafish, although specific NAIP or NLRC4 homologue has not been identified (22).

## Non-canonical inflammasome activation

The mammalian innate immune system senses LPS *via* TLR4, however non-canonical inflammasome activation by intracellular LPS is independent of TLR4 (65). The non-canonical inflammasome results in the activation of caspase-4, -5 or -11, and the non-canonical caspases function as both sensor and effector molecules for LPS. LPS binding induces conformational changes and the oligomerization of caspase-4, -5 or -11. Then the activation of caspase-4, -5 or -11 cleaves GSDMD to induce pyroptosis (66). Furthermore, many studies have shown that IFN inducible GTPases, such as guanylate binding proteins (GBPs), participate in the activation of the non-canonical inflammasome, and facilitate the interaction of LPS with caspase-11. Therefore, GBPs function as critical cofactors for the activation of the noncanonical inflammasome by cytoplasmic LPS (67–69). In addition to LPS, some evidence also supports that the oxidized phospholipid oxPAPC can prevent caspase interaction with LPS and thereby inhibit the non-canonical inflammasome in macrophages (70).

**Figure 3:**
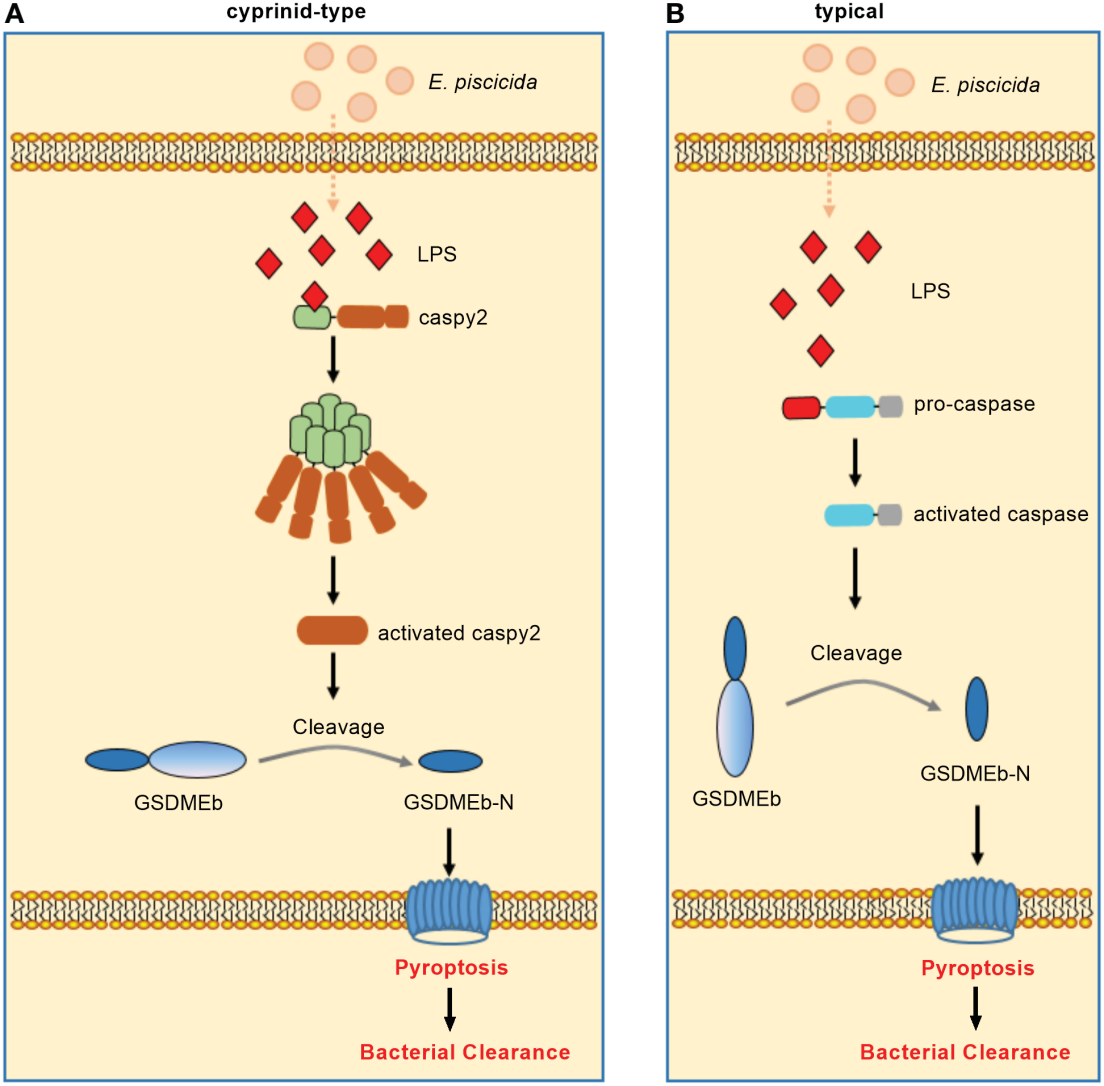
Non-canonical inflammasome activation in teleost fish. **(A)** Non-canonical inflammasome activation in cyprinids. Zebrafish caspy2 binds directly to LPS, resulting in caspy2 oligomerization, which is critical for cleaving GSDMEb to elicit pyroptosis. The caspy2–GSDMEb axis-mediated neutrophil pyroptosisgated NET formation, namely noncanonical NETosis, played a critical role in bacterial clearance. **(B)** Non-canonical inflammasome activation in other fish species. In the turbot, the inflammatory caspase can directly recognize cytosolic LPS, resulting in the GSDMEb cleavage-gated pyroptosis and bacterial clearance.

In zebrafish, caspy preferentially cleaved caspase-1 substrates whereas caspy2 possessed caspase-5–like substrate activity (71). Zebrafish caspy2, but not caspy, directly binds LPS *via* the N-terminal PYD and forms oligomers responding to *E. piscicida* infection, which induces noncanonical inflammasome mediated pyroptosis (23). In addition, three GSDM family proteins, including GSDMEa, GSDMEb and DFNB59, were identified in zebrafish. Similar to mammalian caspase-4/5/11, zebrafish caspy2 can engage the downstream GSDMEb cleavage to gate pyroptosis (72). Furthermore, neutrophils were found to play an important role in bacterial clearance (73). The activation of caspy2 and GSDMEb induced pyroptosis of neutrophils, which was essential for the release of neutrophil extracellular traps (NETs) and the formation of NETs (NETosis). More importantly, the caspy2–GSDMEb axis-mediated NETosis protected the zebrafish from *E. piscicida* infection (24). These findings have led to a model of non-canonical inflammasome activation in zebrafish wherein caspy2 serves as a sensor and effector molecule for LPS (**Figure 3A**).

Interesting, the caspy2 gene is undetectable in other fish databases except zebrafish (74). In addition, most fish species except zebrafish and several other cyprinidae do not exist TLR4. The zebrafish TLR4 does not recognize LPS, which is suggested to be paralogous but not orthologous to mammalian TLR4 (75–77). Since inflammatory caspases have been confirmed to innate immune receptors for intracellular LPS (78), the distinct numbers of identified inflammatory caspases from different fish species (14, 74, 79, 80) suggest that species-specific or diversified functions of inflammatory caspases in the recognition of LPS for inducing noncanonical inflammasome mediated pyroptosis may exist in teleost. For example although little is known about non-canonical inflammasome activation in other teleost fish, a study showed the dual function of an inflammatory caspase (namely SmCaspase) in mediating both canonical and non-canonical inflammasome activation in the turbot *S. maximus* (21). Different from zebrafish caspy and caspy2, SmCaspase contains an N terminal CARD, and a C-terminal large and small subunit of caspase domain. SmCaspase can directly bind with LPS *via* its N-terminal CARD domain, resulting in caspase-5-like enzyme activity-mediated pyroptosis. Furthermore, the SmCaspase-GSDMEb axis-gated pyroptosis controls the bacterial clearance against *E. piscicida* infection (**Figure 3B**). SmCaspase can also be recruited by NLRP3-ASC inflammasome to participate the canonical NLRP3 inflammasome activation (21).

## The functions of inflammasome-associated effectors

Both canonical and noncanonical inflammasome pathways promote pyroptosis, which have been identified as a critical inflammasome effector mechanism. In mammals, the members of gasdermin family proteins, such as GSDMB, GSDMC, GSDMD and GSDME, are cleaved by inflammatory and apoptotic caspases and can result in pore formation (81). However only GSDMD has a pivotal role in inflammasome signaling and pyroptosis, and is the key effector that leads to pyroptosis (81, 82). In the zebrafish genome, only two GSDMEa/b isoforms and a DFNB59 are annotated. Different from GSDMEb, knockdown of GSDMEa could partially rescue microglia cell death in a temperature sensitive *puer* mutant, which harbors a loss-of-function mutation in the NLRC3-like gene (83). In the zebrafish model of tuberculosis, knockdown of GSDMEb can inhibit pyroptosis. Furthermore, zebrafish GSDMEa/b is cleaved by caspy2 for resulting in pyroptosis (18). However in neutrophils, the caspy2–GSDMEb axis but not caspy2–GSDMEa contributed to pyroptosis, NETosis and bacterial clearance (24). Moreover, zebrafish GSDMEa/b also can be cleaved by apoptotic caspases, including caspase 3a, caspase 3b and caspase 7, at the same sites as inflammatory caspases recognized (84). All these data suggest that zebrafish GSDME functions as the equivalent of mammalian GSDMD and GSDME.

**Figure 4:**
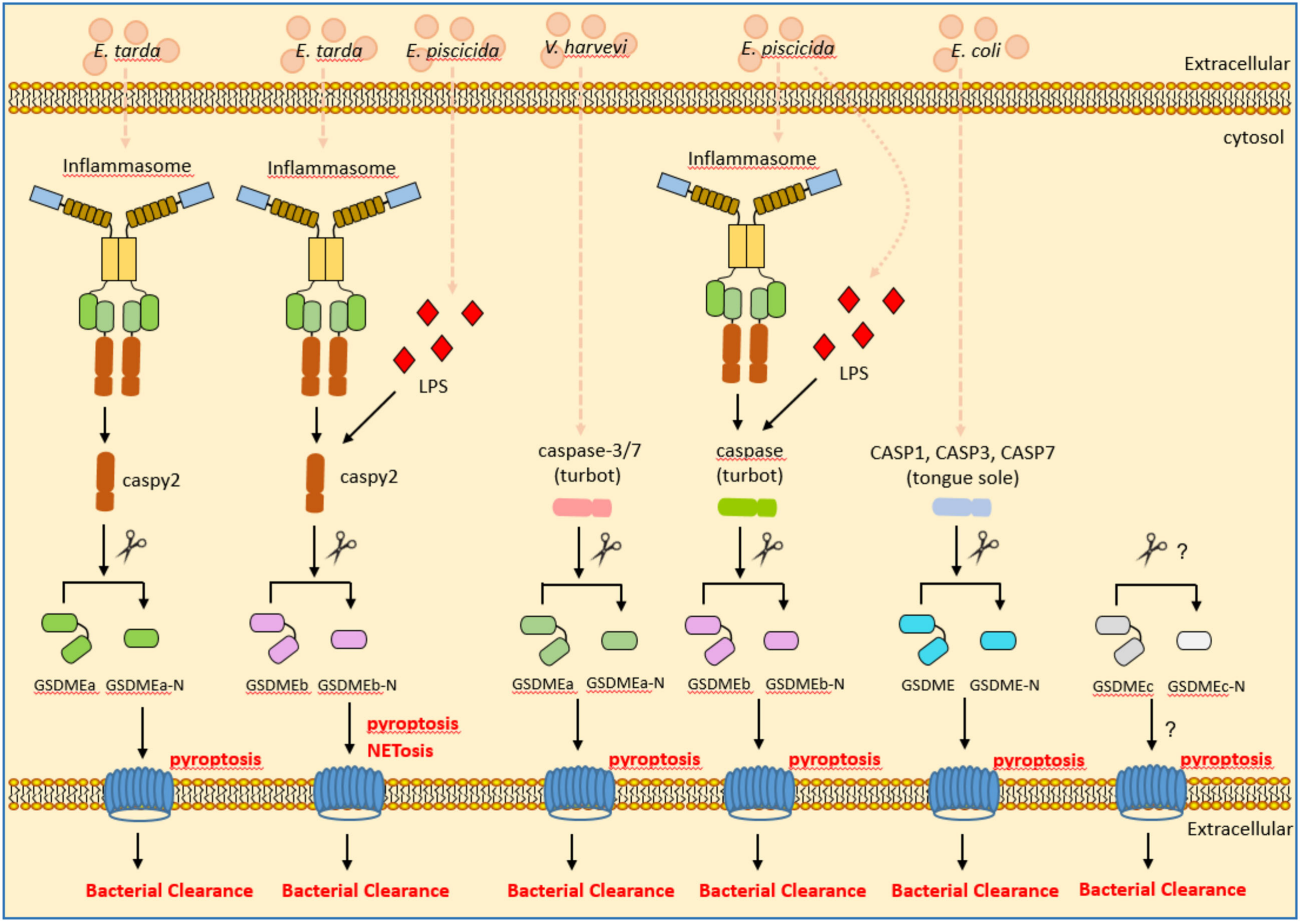
The roles of piscine GSDME in inflammasome signaling, pyroptosis and bacterial clearance. GSDME mediates pyroptosis following cleavage by inflammatory caspases. In the zebrafish and turbot, GSDMEa and GSDMEb were obtained. Zebrafish caspy2 cleaves GSDMEa/b for resulting in pyroptosis and bacterial clearance of *E. tarda* or *E. piscicida*. Turbot caspase-3/7 cleave GSDMEa for resulting in pyroptosis and bacterial clearance of *V. harveyi*. The inflammatory caspase in the turbot cleaves GSDMEb for resulting in pyroptosis and bacterial clearance of *E. piscicida*. In the tongue sole, CASP1, CASP3 and CASP7 cleave GSDME for resulting in pyroptosis and bacterial clearance of *E. coli*.

In addition to zebrafish, GSDME has been characterized in other teleosts including tongue sole (*Cynoglossus semilaevis*) and turbot. In the marine teleost tongue sole, GSDME is cleaved by caspases 1, 3, and 7. Among them, caspase-1-cleaved GSDME induced pyroptosis, whereas caspase-3/7-cleaved GSDME induced switching of cell death from apoptosis to pyroptosis. The cleavage of tongue sole GSDME by caspases 1, 3, and 7 can exert bactericidal activity against *E. coli* (85). Similar to zebrafish, two GSDME orthologues were obtained in the turbot. Although both GSDMEa and GSDMEb can be cleaved by inflammatory caspase, only the turbot GSDMEb can mediate pyroptosis and bactericidal activity against *E. coli* (21). A recent study showed that the turbot GSDMEa is cleaved by caspase-3/7 and caspase-6. The GSDMEa cleaved by caspase-3/7 was activate, and was able to induce pyroptosis and bacterial clearance of *Vibrio harveyi* in turbot. In contrast, the turbot GSDMEa or GSDMEb, which are cleaved by caspase-6 or caspase-8 respectively, were inactivate (86). These findings revealed a regulatory and functional difference of piscine GSDMEs in response to different bacterial infection (**Figure 4**).

Unexpectedly, GSDMEc was identified in perciformes and some primitive ray-finned fishes such as European eel (*Anguilla anguilla*) and reedfish (*Erpetoichthys calabaricus*). However different from GSDMEa and GSDMEb, no obvious caspase recognition site was observed in the linker region of GSDMEc (87). Whether piscine GSDMEc functions in triggering pyroptosis and bacterial clearance remains to be defined.

## Functional roles, regulation and interacting partners of ASC

ASC, which is encoded by the gene PYCARD, is a central component for a number of inflammasomes such as AIM2, NLRP1 and NLRP3 inflammasomes. The polymerization and speck formation of ASC provide a signaling platform with multiple inflammatory caspase binding sites, and the assembly of the ASC speck is critical for inflammasome signaling. Mammalian NLRC3, containing an N-terminal CARD, a central NACHT domain and a C-terminal LRR, could interact with ASC and inhibit the activation of NALP3 and NLRC4 inflammasomes *via* disrupting of ASC speck assembly (88). In mammals, post-translational modifications of ASC also control inflammasome activity. The kinases Syk or Jnk induces the phosphorylation of ASC, which is critical for the formation of ASC specks and inflammasome activity of NLRP3 and AIM2 inflammasomes (89). Furthermore, the linear ubiquitination of ASC by LUBAC affects NLRP3/ASC inflammasome assembly (90). The NLRP3 inflammasome is also activated by TRAF3-dependent ubiquitination of ASC and USP50-mediated deubiquitination of ASC (91). The TRAF6-mediated ASC polyubiquitination leads to the degradation of ASC by autophagy, which suppress the NLRP3 inflammasome (92).

In teleost, the ASC has been identified in zebrafish (17), turbot (93), Japanese flounder (94), goldfish (*Carassius auratus L.*) (95), mandarin fish (*Siniperca chuatsi*) (96), orange-spotted grouper (*Epinephelus coiodes*) (97) and Japanese medaka (*Oryzias latipes*) (98) so on. In many fish species such as zebrafish, mandarin fish, Japanese flounder and turbot, there was only a single ASC gene (99). Several regulatory mechanisms have been revealed for targeting piscine ASC to modulate activities of inflammasomes. In the zebrafish, ASC speck formation leaded to pyroptosis *via* activation of caspy, and the mutation of PYD in the ASC with the impaired functional speck formation abolished NLRP3 inflammasome activity induced by nigericin (17, 100). Homologs of NLRC3 lacking CARD domain or containing other additional domain(s) are classified into NLRC3-like proteins (101).The interaction between NLRC3-like and ASC was confirmed in the goldfish and zebrafish (95, 102). The deficiency of zebrafish NLRC3-like promoted ASC-mediated inflammasome activation and the early control of mycobacterial proliferation (103). Loss of NLRC3-like function resulted in aberrant activation of inflammasome pathway in an ASC-dependent manner. All these data suggest that zebrafish NLRC3-like suppresses activities of inflammasomes by interfering the interactions between ASC and other inflammasome components (83, 102, 103). Interestingly, the analysis from genomes or transcriptomes suggest that the NLRC3 or NLRC3-like expansions exist in different species of fish (15, 101). According to their protein domain compositions, expansive NLRC3 or NLRC3-like proteins in teleost fish fall into at least 8 categories: NLRC3 proteins containing CARD-NACHT-LRR, NLRC3-like proteins containing PYD-FISNA-NACHT-LRR; NLRC3-like proteins containing FISNA-NACHT-LRR, NLRC3-like proteins containing FISNA-NACHT-LRR- SPRY_PRY_SNTX, NLRC3-like proteins containing PYD-FISNA-NACHT; NLRC3-like proteins containing PYD-NACHT; NLRC3-like proteins containing DD-FISNA-NACHT, NLRC3-like proteins containing NACHT-LRR (15, 101). It is not known whether other expansive NLRC3 or NLRC3-like proteins interfere with activities of inflammasomes by targeting ASC. Further studies are required for confirming the roles of those ASC-interacting protein in the regulation of inflammasome activation, especially for NLRC3 or NLRC3-like proteins.

In the Japanese medaka, three ASC (ASC1, ASC2 and ASC3) are tandem replicates on chromosome 16. The large yellow croakers (*Larimichthys crocea*) also have tandem replicate ASC genes. Other fish species such as goldfish and rainbow trout have multiple ASC genes on different chromosomes or scaffolds (99). Although previous studies have shown that the deficiency of ASC1 impairs host defense against *Aeromonas hydrophila* infection and higher expressions of ASC2 and ASC3 were observed in the ASC1-KO medaka than those in the WT, the functional differences between these three types of ASCs are still unclear (98, 99). How these three types of ASCs in teleost fish and their post-translational modifications affect the activation and function of inflammasome need to be further investigated in the future.

## Other sensors or adaptors for assembling the inflammasome complex

In addition to the well-established NLRP1, NLRP3, NLRC4 and AIM2 inflammasome sensors, many other PRRs or sensors such as NLRP6, NLRP7, NLRP12 and Pyrin can assemble the inflammasome complex and regulation of inflammasome activation in mammals (104, 105). The genes coding for AIM2 and Pyrin are not present in teleost (106). Although zebrafish are able to sense cytosolic flagellin and activate an inflammasome complex, the presence of NAIP or NLRC4 homologue in zebrafish was unclear (22). Likewise, most human NLRs involved in inflammasomes do not have one-to-one orthologues in zebrafish. The evolutionary relationship between piscine and mammalian NLRPs is still not resolved (14, 107). It remains to be determined the effects of many other NLR sensors in inflammasome activation in teleost fish.

In addition to the interferon (IFN)-induced AIM2 and Pyrin, IFN-induced GTPases are also involved in inflammasome activation in mammals (108, 109). The 65–73 kDa GBPs comprise a family of highly conserved IFN-induced GTPase family, 7 GBPs in human and 11 GBPs in mouse (110). There are several studies regarding GBPs in teleost fish. The first characterization of GBPs in fish was described by Robertsen and colleagues in 2006, who found that trout GBP showed expression properties similar to mammalian GBPs (111). Interesting, the loss of GBP genes was observed in the pufferfish (*Tetraodon nigroviridis*), fugu (*Takifugu rubripes*), stickleback (*Gasterosteus aculeatus*) and medaka (112). In zebrafish, 8 GBPs have been found, with the GBP1 and GBP4 being studied. Zebrafish GBP4 has a similar architecture as GBP1, with an N-terminal GTPase domain, a helical domain and a C-terminal CARD (113, 114). The GTPase activity of zebrafish GBP4 is crucial for the activation of inflammatory caspy, and indispensable for inflammasome activation and bacterial clearance. Unexpectedly, zebrafish GBP4-dependent clearance of intracellular *S. Typhimurium* by neutrophils and caspy activation are dependent on bacterial flagellin and ASC (114). The roles of other GBPs in mediating inflammasome activation in response to bacterial infection are still unclear in teleost fish. Since the numbers of GBP numbers vary on the different fish species and certain GBPs may not appear to have a role for inflammasome activation, further studies are warranted to investigate why certain GBPs can target bacteria to drive inflammasome activation and others do not?

ASC is a common adaptor protein for most inflammasomes. However, the Caiap is a new inflammasome adaptor in teleost fish. Using a PFAM search to identify proteins harboring CARD domains, the Caiap with an N-terminal CARD and 16 C-terminal ANK repeats was identified in the zebrafish. The orthologs of zebrafish Caiap exists in amphibian, reptiles, birds and marsupials, but not in placental mammals. Caiap functions downstream of flagellin and interacts with the active caspy *via* its ANK repeats but not with ASC. Strikingly, the CARD domain of zebrafish Caiap allows its self-oligomerization and mediate the inflammasome-dependent resistance to *Salmonella enterica* serovar Typhimurium (115). However whether piscine Caiap interacts with inflammasome sensors has yet to be identified.

## Inflammasomes in innate immune responses

Innate immunity mediated by PRRs provides the first line of defense against invading pathogens. However, some PRRs form inflammasomes for producing inflammatory cytokines and inducing pyroptotic cell death (2). Inflammasomes themselves are also members of the innate immune system, and further regulate innate immune responses after activation. Macrophages and neutrophils are critical for innate immune responses. Macrophage death by pyroptosis due to the activation of the NLRC4/caspase-1 inflammasome prevents the replication of intracellular pathogens, however bacterial clearance depends on the subsequent activation of neutrophils in this case (116–119). In the absence of downstream killing by neutrophils, pyroptosis can increase bacterial dissemination and cause significant damage to host tissues (118, 119). Furthermore, the NLRP3 and AIM2 inflammasomes can affect macrophage polarization. The activation of NLRP3 inflammasome promotes proinflammatory M1 macrophage polarization, whereas the activation of AIM2 inflammasome reversed the phenotype from anti-inflammatory M2 to pro-inflammatory M1 (120, 121). In NLRP3^−/−^ mice, more severe injury and inflammation were observed in response to dextran sulphate sodium (DSS)-induced colitis. Functional responses to bacterial MDP was lacking in macrophages isolated from NLRP3^−/−^ mice, the impaired chemotaxis and enhanced apoptosis found in neutrophils isolated from NLRP3^−/−^ mice (122).

The pyroptosis of macrophages and neutrophils can also be triggered by the noncanonical inflammasome, which confers host defense against pathogen infection. In response to cytosolic bacteria such as *B. pseudomallei* and bacteria that aberrantly enter the cytosol such as *S. typhimurium* mutant or *Legionella pneumophila* mutant, macrophages activate caspase-11 to initiate pyroptosis independent of all known canonical inflammasomes, and play critical roles in limiting these bacterial infection (123). In response to extracellular pathogens, NETs made by activated neutrophils degrade virulence factors and kill pathogens (124, 125). Activated caspase-11 cleaves GSDMD and triggers neutrophil pyroptosis. GSDMD-dependent neutrophil death evokes the extrusion of antimicrobial NETs, which prevent bacterial dissemination and also protect neutrophils from pathogen invasion of cytosolic *S. typhimurium* mutant ΔsifA (126).

In teleost, only several studies reveal the roles of piscine inflammasomes in innate immune responses. Dysregulated haemolysin of *E. piscicida* were found to mediate pyroptotic-like cell death *via* noncanonical inflammasome signaling, which in turn enhance host defence for restricting bacterial colonisation in zebrafish intestine by initiating inflammation (127). During the infection of hemolysin-overexpressing *E. piscicida* (EthA^+^), the caspy2–GSDMEb axis induced pyroptosis of neutrophils, which contributed to NETosis and bacterial clearance *in vivo* (24). In response to the infection of Lm-pyro *L. monocytogenes* strain, the activation of inflammasome similar to the murine NAIP5/NLRC4 inflammasome in zebrafish induces macrophage recruitment to infection sites. Both macrophages and neutrophils are important for bacterial clearance, however only macrophages are essential for controlling *L. monocytogenes* infection in the context of inflammasome activation (22). In summary, emerging data point to the importance of cellular immune responses to piscine inflammasome activation in control of invading pathogens.

## Conclusions and future perspectives

Although the canonical and non-canonical inflammasome pathways, as well as the mechanism and role of inflammasome activation and subsequent pyroptosis have been elucidated in teleost fish, current research on fish inflammasomes is far from enough, and most studies only focus on zebrafish model organism. Compared with the mammals, the inflammasome-dependent functions of NLRs remain poorly defined. The expansions of piscine NLRs suggest that inflammasome components may be much more diverse than thought. However the identified canonical inflammasomes in fish are mainly NLRP1 and NLRP3. Future studies are suggested to reveal the role of other NLR inflammasomes or other NLR sensors in inflammasome activation. Furthermore, there may be multiple inflammasome components or inflammasome-associated effectors with different structures or activities, depending on the fish species selected. In particular, the structure of inflammatory caspase differs between most fish and cyprinids. The effects of caspases on GSDMEs differentiate in different fish species. The specific regulatory mechanisms of inflammasomes and activation mechanism of inflammasome-associated effectors remains to be further investigated in more fish species and in the case of pathogen infection. A better understanding of the relationship between inflammasome activation and pathogen clearance in teleost fish will reveal new molecular targets for treatment of inflammatory and infectious diseases in aquaculture.

## Author contributions

MC conceived, wrote and revised the manuscript. The author confirms being the sole contributor of this work and has approved it for publication.
